# Supplementary material for: Comparative multiomics analysis of cell physiological state after culture in a basket bioreactor
Source: Sci Rep. 2022 Nov 23;12:20161. doi: 10.1038/s41598-022-24687-4 (PMC9686226; doi:10.1038/s41598-022-24687-4)
Supplement: Supplementary file 1 — Supplementary Information 1. [file 41598_2022_24687_MOESM1_ESM.zip › raw data/Proteomic raw data/6.KEGGenrichment/EG.vs.CG/DOWN/pathway/src/04014.html]

map04014


- K04357, K08774, K18496, K18497, K04358, K05451, K02582, K04355, K04356, K12457, K04526, K05459, K13769, K04359, K17386, K05450, K05453, K05461, K05454, K05448, K16858, K16859, K05449, K05460, K05465, K05466, K05467, K05462

- Down regulated proteins


A0A0D9S6I0(Description:Macrophage colony-stimulating factor 1;Gene:CSF1;log2FC:-1.2197)

- K13618, K01047, K16342, K16343, K16817

- Down regulated proteins

A0A0D9R3Z2(Description:Lipase domain-containing protein;Gene:PLA1A;log2FC:-inf)

Close
